# BioPatRec: A modular research platform for the control of artificial limbs based on pattern recognition algorithms

**DOI:** 10.1186/1751-0473-8-11

**Published:** 2013-04-18

**Authors:** Max Ortiz-Catalan, Rickard Brånemark, Bo Håkansson

**Affiliations:** 1 Department of Signals and Systems, Biomedical Engineering Division, Chalmers University of Technology, Gothenburg, Sweden; 2 Centre of Orthopaedic Osseointegration, Department of Orthopaedics, Sahlgrenska University Hospital, Gothenburg, Sweden

**Keywords:** Prosthetic control, Pattern recognition, Artificial limbs, Myoelectric signals, EMG, Regulatory feedback networks

## Abstract

**Background:**

Processing and pattern recognition of myoelectric signals have been at the core of prosthetic control research in the last decade. Although most studies agree on reporting the accuracy of predicting predefined movements, there is a significant amount of study-dependent variables that make high-resolution inter-study comparison practically impossible. As an effort to provide a common research platform for the development and evaluation of algorithms in prosthetic control, we introduce BioPatRec as open source software. BioPatRec allows a seamless implementation of a variety of algorithms in the fields of (1) Signal processing; (2) Feature selection and extraction; (3) Pattern recognition; and, (4) Real-time control. Furthermore, since the platform is highly modular and customizable, researchers from different fields can seamlessly benchmark their algorithms by applying them in prosthetic control, without necessarily knowing how to obtain and process bioelectric signals, or how to produce and evaluate physically meaningful outputs.

**Results:**

BioPatRec is demonstrated in this study by the implementation of a relatively new pattern recognition algorithm, namely Regulatory Feedback Networks (RFN). RFN produced comparable results to those of more sophisticated classifiers such as Linear Discriminant Analysis and Multi-Layer Perceptron. BioPatRec is released with these 3 fundamentally different classifiers, as well as all the necessary routines for the myoelectric control of a virtual hand; from data acquisition to real-time evaluations. All the required instructions for use and development are provided in the online project hosting platform, which includes issue tracking and an extensive “wiki”. This transparent implementation aims to facilitate collaboration and speed up utilization. Moreover, BioPatRec provides a publicly available repository of myoelectric signals that allow algorithms benchmarking on common data sets. This is particularly useful for researchers lacking of data acquisition hardware, or with limited access to patients.

**Conclusions:**

BioPatRec has been made openly and freely available with the hope to accelerate, through the community contributions, the development of better algorithms that can potentially improve the patient’s quality of life. It is currently used in 3 different continents and by researchers of different disciplines, thus proving to be a useful tool for development and collaboration.

## Background

Processing and pattern recognition (PatRec) of bioelectric signals have been at the core of prosthetic control research in the last decade [1,2]. Researchers have employed a wide variety of algorithms aiming to improve the controllability of prosthetic devices, and although most of them agree on reporting the accuracy of predicting movements, there is a significant amount of study-dependent variables that hinder high-resolution inter-study comparisons. Examples of such variables are: electrode type, size, and placement; amplifiers, filters, and acquisition hardware specifications; signals segmentation and characterization; and, protocols for the acquisition of the bioelectric signals.

As an effort to provide a common research platform for the development and evaluation of algorithms in prosthetic control, BioPatRec is introduced as open source software in this work. BioPatRec is a modular platform implemented in Matlab [3] that allows a seamless integration of a variety of algorithms in the fields of: 

1. Signal processing

2. Feature selection and extraction

3. Pattern recognition

4. Real-time control (control engineering)

BioPatRec includes all the required functions for myoelectric control; from data acquisition to real-time evaluations, including a virtual reality environment and pattern recognition algorithms. Moreover, BioPatRec functionalities are easily available through graphical user interfaces (GUIs) in order to facilitate utilization.

In this work, BioPatRec is demonstrated through the implementation of a relatively new paradigm in pattern recognition, namely Regulatory Feedback Networks (RFN). RFN herein is compared with two of the most popular pattern recognition algorithms in prosthetic control: Multi-layer Perceptron (MLP) and Linear Discriminant Analysis (LDA). Although the offline performance of MLP and LDA have been compared previously [4-6], this is the first time they are benchmarked using a real-time evaluation. Additionally, demonstrations of BioPatRec used for the real-time control of a virtual hand, and multifunctional prosthetic devices, are provided.

In the field of machine learning, a common practice is to compare algorithms using the same data sets. This is not the case in prosthetic control, where only few studies have compared more than 2 algorithms under the same settings [4,6,7]. Conducting research based on the scientific method demands repeatability. BioPatRec not only offers a common evaluation platform, but also a publicly available repository of myoelectric signals (MES) to allow high-resolution comparisons and algorithms benchmarking.

Institutions with tradition in myoelectric control such as the University of New Brunswick (UNB) and the Rehabilitation Institute of Chicago (RIC), among others, have developed similar software platforms along their years of research. The Classifier Evaluation in a Virtual Environment (CEVEN) from UNB was one of the first programs that used a virtual reality environment for testing and evaluating prosthetic control [8], as well as software independently developed at Lund University [9]. UNB also produced the Acquisition and Control Environment (ACE) [10] which control functionalities were used together with the MusculoSkeletal Modeling Software (MSMS) [11] to produce the Virtual Integration Environment (VIR) [12]. This was part of The Revolutionizing Prosthetics 2009 project sponsored by the Defense Advanced Research Project Agency (DARPA) in USA. More recently, RIC developed its own and extended research platform, Control Algorithms for Prosthetics System (CAPS), which has been used to pioneer tests for real-time evaluation [13,14]. These are all modular and sophisticated platforms that allow the investigation of different myoelectric control strategies, mainly based in pattern recognition. Unfortunately, their accessibility is limited since they are proprietary and therefore only internally available. To our knowledge, there is currently not a complete research platform devoted to prosthetic control based in pattern recognition which is neither open-source, nor proprietary but publicly available on licensing basis.

Collaboration through different fields was a driving factor to open source BioPatRec. Since BioPatRec is a highly modular and customizable platform, researchers from different fields can seamlessly benchmark their algorithms by applying them in prosthetic control. For example, an A.I. specialized researcher can easily add a pattern recognition algorithm without necessarily knowing how to obtain and process bioelectric signals, or how to produce and evaluate physically meaningful outputs. In the same way, a control researcher could implement control algorithms without worrying about the implementation of classifiers. It is worthy of notice, that the aim of BioPatRec is not to obscure any of these fields but to ease their integration.

## Methods

### BioPatRec implementation

BioPatRec is implemented as a collection of functions and GUIs divided in the following modules: 

•Signal Recordings

•Signal Treatment

•Signal Features

•Pattern Recognition

•Control

**Figure 1:**
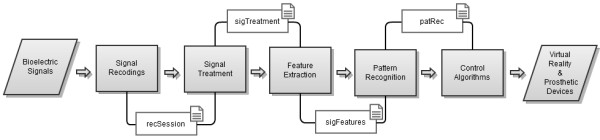
**BioPatRec flow diagram.** BioPatRec is organized in different modules that are linked through the use of structure arrays. These structure arrays can be saved and loaded between the different modules. This also allows replacing or modifying any module without affecting the others, given that the structure arrays are preserved.

BioPatRec’s modular architecture is linked by structure arrays that enable the communication between the different modules (see Figure 1). The first open source release, “BioPatRec ETT”, is presented in this work and further referred as “BioPatRec” only.

These structure arrays allow the modification, enhancement, or replacement of any module without affecting the others, thus providing great flexibility for implementing new algorithms. Moreover, BioPatRec has a user friendly design with GUIs that allow easy customization of different experiments. It also includes a considerable amount of supporting routines aiming to reduce developing time and allow the user to focus on specific experiments. A summary of BioPatRec features is given in the Additional file 1.

All the required instructions for use and development are provided in the online project hosting platform (http://code.google.com/p/biopatrec) [15]. This freely available site includes issue tracking and an extensive “wiki”, where a considerable amount of information has been documented, and can be continuously updated by the community. The transparent implementation aims to facilitate utilization, but more importantly, collaboration.

#### Recording of bioelectric signals

Signals acquisition can be performed in three different ways to serve different purposes. One-shot recordings. These are fixed-time real-time displayed recordings mainly use to verify the correct functioning of the acquisition hardware, as well as for inspecting the signals quality. Problems of lead failure, electrode positioning, and interference can be easily identified by observing the signals recorded in real-time. Recording Session. During a recording session, the user is instructed to perform preselected movements guided with different visual cues, such as images and progress bars. The settings of the recording sessions such as sampling frequency; acquisition hardware and arbitrary channels selection; contraction duration as well as relaxation, in between others, are easily defined using a dedicated GUI. The recording session produces the structure array *recSession* which can be later loaded and displayed for examination. Recordings for real-time control. The settings used in the recording session are kept through the different modules in order to be reproduced when required in the real-time control.

BioPatRec is released with data acquisition routines on the Session-Based Interface (SBI) paradigm. SBI allows a wide variety of data acquisition hardware to use the same routines. The SBI has been tested for the USB-6009 and USB-6212 data acquisition cards (National Instruments, Austin, USA). Additionally, acquisition routines using the Serial Computer Interface (SCI) to communicate with microcontrollers are also available.

#### Signal treatment

**Figure 2:**
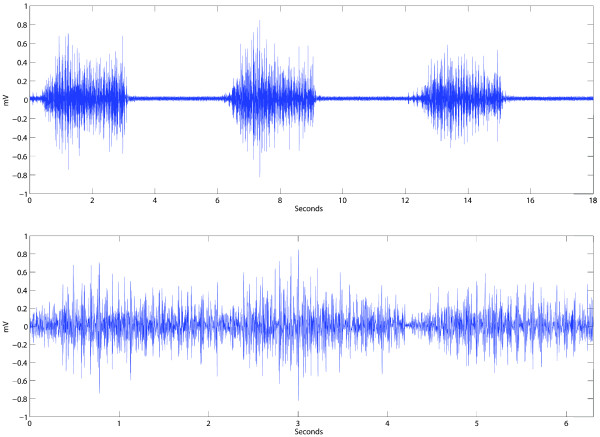
**Signal processing: contraction time percentage (cTp).** The top figure shows a single channel of a recording session that requested the repetition of a given movement 3 times with 3 seconds contraction time, and equal resting periods. The bottom figure shows the same signal trimmed to 70% of the contraction time. During signal treatment, the total of the recorded signal is segmented to extract the periods of interest. cTp can be used to include or remove transient periods.

The recording session aims to capture as much information as possible on the intended movements. In contrast, the signal treatment routines aim to reduce this information to a more optimal form for pattern recognition. Through a dedicated GUI, channels and movements of no interest for specific studies can be easily removed. The absences of movement, or resting condition, can be automatically added as an additional movement using the signals of the resting periods in the recording session. The signals recorded during the contraction time can be trimmed to exclude the transient period of the contraction (isotonic). This is achieved by selecting the contraction time percentage (cTp) which limits the portion of the myoelectric signals that characterize each movement. Figure 2 shows one channel of a recording session which is later processed with 70% cTp. Full cTp would most likely capture periods without any movement, while 50% cTp would mostly consist of the isometric part of the contraction. The signal is trimmed equally at the beginning and ending of the contraction time.

**Figure 3:**
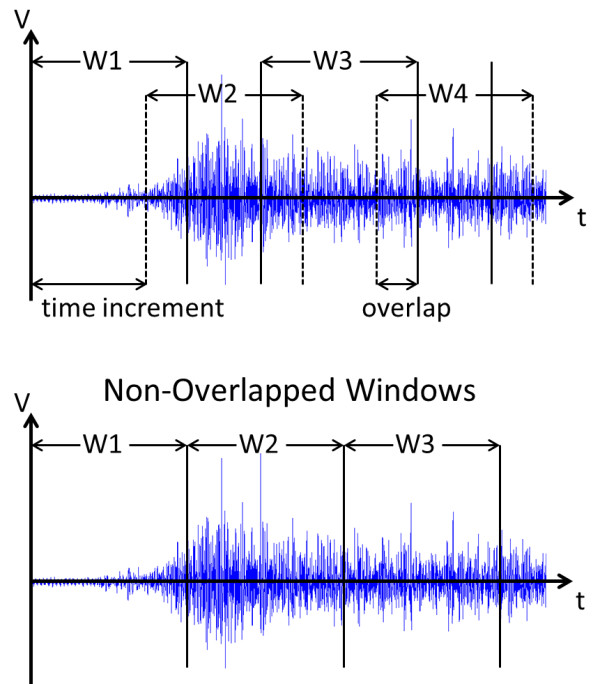
**Signal processing: time window.** Myoelectric signal segmentation by time windowing including overlap or non-overlapped segments.

Additionally, different frequency and spatial filters are available. Frequency filters such as to reduce the power line harmonics (PLH) or Butterworth band-pass at different frequencies are implemented, as well as single and double differential spatial filters for special electrode arrangements. The last part of signal processing in this module takes care of the signal segmentation by overlapping and non-overlapping windowing, see Figure 3. This also includes the size selection for the training, validation and testing sets.

#### Signal features

**Figure 4:**
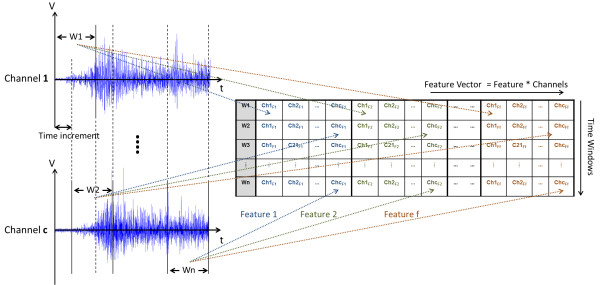
**Signal features: feature vectors.** Construction of the feature vectors (FVs) from bioelectric signals recorded during the execution of a given movement. Example of “f” features extracted from “c” channels, and “n” time windows (“W”). The FV is composed by the extracted signal features in a specific time window from all channels. There are as many FVs as windows for a given movement.

Although few pattern recognition algorithms can receive time series as input, the vast majority require a discretized characterization of the signal, commonly known as signal features, see Figure 4. These can be statistical descriptors such as the mean absolute value, or more sophisticated measurements such as fractal dimension or rough entropy. A wide variety of signal features have been historically used in prosthetic control [16], unfortunately with no generalized consensus on which feature, or set of features, provide the best characterization, see Table 1. It is worthy of notice that apparent popularity of the most commonly found sets in the prosthetics pattern recognition literature, is due to the large influence on the field of two research groups (UNB and RIC), which does not necessarily mean that these sets are the most widely used for the entire research community.

**Table 1:** Non-exhaustive compilation of myoelectric signal features employed in pattern recognition for prosthetic control

	**Publication references**														
	[6,14,17-25]	[26]	[27]	[7]	[5,28]	[29]	[30]	[31]	[32]	[33]	[34,35]	[36]	[37]	[38]	BioPatRec*
Mean absolute value	x	x	x	x	x	x	x	x	x	x	x				x
Zero crossing	x	x	x	x	x	x	x								x
Slope sign changes	x	x		x	x	x	x								x
Waveform length	x	x	x	x	x	x	x	x		x					x
Mean abs. value slope		x	x												x
Root-mean square				x	x	x		x	x			x			x
Variance				x				x							x
William amplitude				x											
Wavelet transf.			x												
Wavelet packet transf.			x												
Fourier transf.			x												x
Autoregression coeff.					x		x								
Integral abs. value						x									
Sample skewness						x									
Fractal dimension								x							x
Max. fractal length								x							x
Quadratic polynomials													x		
Rough Entropy														x	x

* Non-exhaustive list of features included in the “ETT” release. See the online project for all features [15].

BioPatRec is released with 27 signals features in time and frequency domains that can be used to feed pattern recognition algorithms. The feature extraction routines are implemented in a way that the inclusion of new features can be simply done by adding an identifier, and then naming the computation routine accordingly. Detailed instructions are provided in the online hosting platform [15], or can be easily deduced from the code. Additionally, commonly used sets of features can be directly selected in the GUI for pattern recognition.

The signal processing and feature extraction routines are called from the same GUI, although divided by two different data structures (*sigTreated* and *sigFeatures*, see Figure 1). This makes it possible to separate them if needed. Additionally, a function has been implemented to treat a series of recording sessions with the same signal processing and feature extraction settings (*Treat Folder*). This BioPatRec feature aims to facilitate further evaluation of pattern recognition in large groups of subjects.

#### Pattern recognition

The pattern recognition module is divided in Offline and Real-time classification. The utility of having separated processes is notably during the implementation of new algorithms, where testing and benchmarking is simplified by only using recorded sessions. It is also necessary when acquisition hardware or testing subjects are not available.

**Figure 5:**
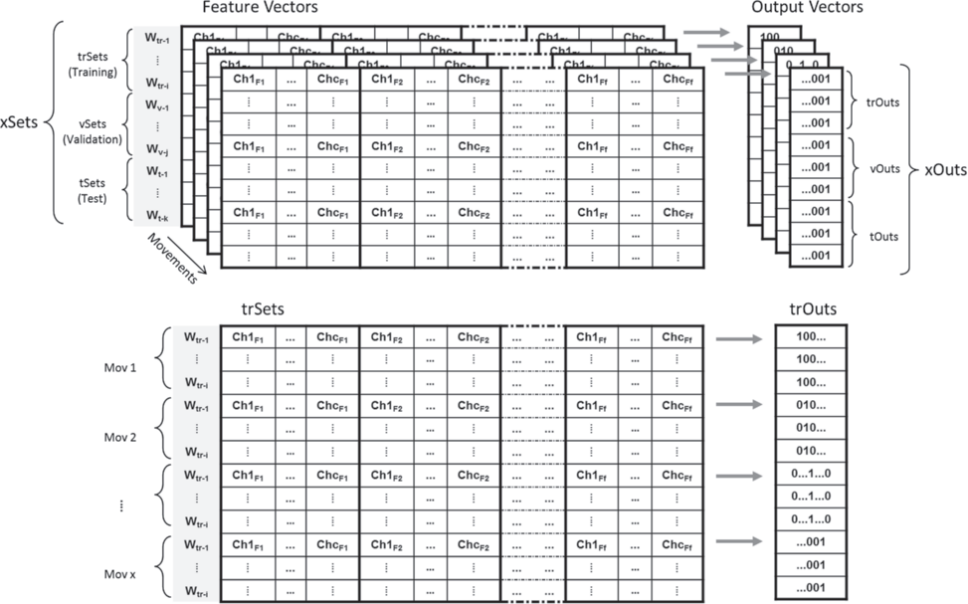
**Pattern recognition: The xSets.** The xSets, and corresponding xOuts, are the ensemble of data sets to be used in the different stage of offline pattern recognition. xSets and xOuts are ultimately 2 dimensional matrices where sets of a given phase, e.g. training, are stack over each other. Once all movements are merged, they can be distinguished by the xOuts matrices.

The Offline PatRec has been implemented in 3 phases: training, validation, and testing. Pre-recorded myoelectric signals (*recSession*) are used to create independent data sets, or feature vectors, which are assigned to each of these phases, see Figure 5. The training and validation sets are meant to be used during the learning process. Contrarily, the testing sets are only used once the classifier has been trained to evaluate its performance with unseen data.

Traditionally, there is ambiguity in the understanding of each of these steps due to the different nature of each pattern recognition algorithm. However, although they might not be literally correct for all algorithms, they provide a general framework for implementation. For example: Although RFN does not require of a formal training phase, its connectivity matrix must be calculated before the classifier can be used. On the BioPatRec’s framework, this computation could be understood as the “training”, and since it can be computed in different ways, the *Training algorithm* field can be used to discriminate between the differing computational options.

The real-time routines require a classifier (*patRec*, see Figure 1) trained in the offline step which contains all the relevant information to reproduce the pattern recognition, such as the data acquisition settings and signal processing methods. Real-time PatRec delivers constant predictions of intended movements, which can be used for controllability evaluations. A measure of real-time performance is normally lacking in the literature, despite that it has been shown to be required to truly evaluate controllability [8]. Therefore, BioPatRec includes two real-time tests that provide more realistic evaluations of the clinical utility of a given control strategy. The Motion Test introduced by Kuiken *et al.*[13], consists of demanding the subject to execute the trained movements in a random order, while evaluating the following key performance indicators: The Target Achievement Control (TAC) test is a step closer to reality from the motion test. Although it requires a virtual reality environment which limits its availability, it enhances the control strategy evaluation by simulating a prosthetic device. Introduced by Simon *et al.*[14], it employs the same key performance indicators as the motion test. Two virtual limbs are displayed to the user; one shows the target position while the other is controlled by the user departing from a neutral posture. Two important features of the TAC test are: 1) the target position is never at the end of the posture which allows the user to overshoot the position; 2) misclassification has now a more realistic impact by deviating the motion from its target. Both of these situations would require the user to compensate with ago-antagonistic movements, as in the real scenario. Finally, the target position must be hold for a predefined amount of time to be considered as a completed motion. The TAC test is a recently added feature to BioPatRec currently under evaluation but available in the release (BioPatRec ETT).

• **Selection time.** It measures the time required for the controller to produce the first correct prediction, therefore it can be seen as an indication of responsiveness. It starts immediately before the first prediction different to “rest” or “no movement”. In the BioPatRec implementation, it is also included a time window required for extracting the signal features, as well as the computation time required for signal processing and classification.

• **Complementation time.** It is intended as a stability indicator that accounts for the time required to achieve 20 correct predictions using the same starting timestamp as for the selection time. Similarly to the selection time, it includes the length of the first time window additionally to the computation time required for processing and classification. In the original implementation by Kuiken *et al.*[13], only 10 predictions were used, however, we have empirically found that 10 predictions were easily achieved during 5 seconds in our experimental setup, even by chance. Therefore, the predictions required to consider a completed motion was raised to 20, which we found harder to achieve without perceivable stability. It is worthy of notice, that the prediction speed depends considerably on the processing hardware, therefore the number of predictions used might vary in different systems. In our setup, a new prediction was made every 50 ms.

• **Completion rate.** It refers to the number of requested movements that achieved completion time within the time deadline.

• **Real-time accuracy.** During experimental trials, it was found that the completion time alone was not enough to reflect the stability of the controller since it depends considerably on the processing hardware. Therefore, the prediction accuracy during the completion time was also introduced. For exmaple, if the completion time took 25 time windows, thus producing 25 predictions from which 20 were correct, the prediction accuracy would be 80%.

#### Pattern Recognition Algorithms (PRAs)

BioPatRec can easily integrate different PRAs and it is initially released with 3 of them, each of a different nature. For an updated list of available algorithms, as well as details on the implementations, see the online project [15]. Linear Discriminant Analysis (LDA). Discriminant Analyses (DA) are statistical methods for pattern recognition which fundamentally relates to the analysis of variance. As directly available from Matlab, 5 types of DA can be used: linear, linear with diagonal covariance matrix, quadratic, quadratic with diagonal covariance matrix, and Mahalonbis [39]. Algorithms based in Linear Discriminant Analysis (LDA) have been used considerably in prosthetic control due to simplicity, speed and accuracy [4,7,13,14,17,18,40]. LDA finds a linear transformation, or discriminant function, that separates the data by minimizing the inter-class distance and maximizing the intra-class distance. In other words, it tries to find a linear combination of the features that characterized each signal, thus separating them into different groups. Although LDA performs dimensionality reduction, it differs from Principal Component Analysis (PCA) by focusing on the data itself rather than features, thus preserving most of the discriminant information. Multi-layer Perceptron (MLP) is a feedforward topology of Artificial Neural Networks (ANNs). ANNs are inspired by their biological counterpart and have applications beyond pattern recognition such as control engineering. The ANN’s outputs depend on the weight assigned to the connection of each neuron. Even though it has been proved very useful to solve several problems in classification and prediction, their main drawback is that the network design is very experimental, for more details in MLP and ANN see [41]. The BioPatRec implementation uses the logistic (sigmoidal) activation function, and allows customizable hidden layers and neurons in each hidden layer. The training could be performed by batch, or stochastically in a given percentage of the training sets. Additionally, the detection of poor convergence to automatically reset the training is available. MLP is a stand-alone implementation for BioPatRec which does not require additional toolboxes. Regulatory Feedback Networks (RFN). Traditionally, pattern recognition is performed by training a classifier (training phase) which can later make predictions on the learned classes by looking at similar input data (testing phase). It is therefore intuitive that most of the attention is paid to the learning processes in comparison with the testing phase. Conversely, RFN requires no formal learning, or modification of its connectivity matrix (weights) during a training process [42]. Originally introduced as Input Feedback Networks by Achler [43], RFN predictions occur directly in the testing phase through network outputs top-down self-inhibition, or negative feedback as better known from control theory. The future state of any feedback dependent system is given by the current inputs and the processed outputs. Given a connectivity matrix *W*_*i*,*j*_, where *j* represent the features per class *i*, and considering *Y*_*a*_, a system output of index *a*, the future state of *Y*_*a*_ is updated according to the overall activity of its inputs *I*_*j*_, and its class representation in the connectivity matrix.

(1) $$Y_{a}(t+\mathrm{\Delta t})=\frac{Y_{a}(t)}{n_{a}}\overset{N_{a}}{\underset{j=1}{\sum }}I_{j}*W_{a,j}$$

where *N*_*a*_ denotes the inputs projecting to *Y*_*a*_, and *N*_*a*_ is the normalization value accounting for the processes in set *N*_*a*_.

(2) $$n_{a}=\overset{N_{a}}{\underset{j=1}{\sum }}W_{a,j}$$

The salience of input *I*_*j*_ is regulated by the feedback from neurons which it projects to (*Q*_*j*_), and it is driven by the raw input data (*X*_*j*_).

(3) $$I_{j}=X_{j}/Q_{j}$$

The shunting inhibition corresponds to the sum of the activity of all neurons *Y*_*i*_ receiving activation from *I*_*j*_.

(4) $$Q_{j}=\overset{M_{b}}{\underset{i=1}{\sum }}Y_{i}(t)*W_{i,j}$$

where *M*_*b*_ denotes the feedback connections to input *I*_*j*_. The general RFN model and the stability of its equations are analyzed in [42].

In the case of prosthetic control, the representation of a class is traditionally given in a set of feature vectors extracted from several time windows, see Figure 4. In order to construct the connectivity matrix, these vectors can be averaged to form a single feature vector per class. Additionally, since no learning is required and each output inhibits only its own inputs, new classes can be added directly without modification of the established connectivity matrix, besides the addition of the new vector of features. This characteristic also prevents catastrophic failure (forget previously learned classes). Normalization is usually required to avoid that features with large magnitudes eclipse the contribution of the rest. Different normalization methods are included in BioPatRec, such as the statistical normalization (*μ*=0 and *σ*=1), unitary range (0 to 1), and 0-midrange with 2-range (-1 to 1). The choice of the normalization method depends strongly on the implementation of a given algorithm, and it can greatly affects the classifier performance. For example, we have empirically found that randomly initializing the MLP’s weights between -1 to 1, and normalizing the inputs into the same range, reduce the training time and improves convergence, as suggested by [41].

#### Control

Control strategies or post-processing algorithms can be applied to the output of the classifier in order to considerably improve the real-time stability of the system. BioPatRec is initially released with two algorithms: 

• **Majority voting.** Sporadic misclassification can be filtered by this algorithm which employs a recent history buffer of predicted movements. At any time, the movement which has the most active presence in the buffer is considered as the “winning” output. The stability provided by this algorithm comes at the cost of slower response since a given number of predictions are required for the buffer.

• **Buffer output.** Since majority voting is inherently inappropriate for simultaneous control (see future work), an alternative, but similar strategy, is to employ thresholds to decide if a given output has been selected enough to be considered as a correct classification. The threshold is set to a given percentage of presence in the buffer. In this strategy, outputs do not compete with each other but simply need to be produced consistently to be correct.

Besides the utility of these algorithms, which will be evaluated in future studies, they have been released to provide a framework where other more sophisticated strategies can be implemented.

#### Matlab

Although BioPatRec has been developed in Matlab [3] which is a proprietary software, it is also a widely available and well-known tool in the academic and research community. Matlab has several easy to use and powerful mathematical libraries/toolboxes that facilitate the implementation of algorithms, thus reducing development time. Additionally, projects in Matlab are easily transferred within the platform, which in turn facilitates collaboration. Examples of related developments can be found in the Myoelectric Control Development Toolbox, a set of isolated routines for myoelectric control [44]; and The BioSig project, an open source library for bioelectric signal processing [45]. Open sources projects on pattern recognition such as NETLAB [46], The Bayes Net Toolbox [47], and The WaveAtom Toolbox [48], also use Matlab [3] as platform.

### Repository of recording sessions

The common repository of bioelectric signals enables experiment reproducibility and high-resolution comparison. It also allows further studies to take place on data sets which potentially contain more information than what can be examined in a single study. The bioelectric signals are contained together with all the relevant information of the recording session in a structure variable (*recSession*), which can be easily shared or exported/imported into other programs.

A set of recording sessions from 17 non-amputee subjects are provided under the label “10mov4chUntargetedForearm”. These correspond to 4 differentially recorded myoelectric signals digitalized at 2 kHz with a 14-bits resolution. The use of 4 bipolar electrodes has been proved to be sufficient for the classification of at least 10 hand and wrist movements [17,49]. The electrode placement was untargeted but equally spaced around the forearm proximal third. The first pair (channel 1) was consistently placed along the extensor carpi ulnaris, and the rest following the radius direction. The proximal electrode was always connected to the positive terminal of the biopotential amplifier. It has been shown that offline accuracy over 95% can be reached using 4 electrodes either selectively or symmetrically placed [4]. The untargeted placement, equivalent to symmetrical in this context, is more practical in the clinical settings, thus motivating the development of algorithms that are robust under these circumstances. Furthermore, it has been shown that classification accuracy is more sensitive to electrode shifts when using selective placement [50].

The biopotential amplifier was an in-house design (MyoAmpF2F4-VGI8) with a variable gain up to 74 dB (set to 71 dB at 300 Hz), and embedded active filtering: 4th order high-pass filter at 20 Hz; 2nd order low-pass filter at 400 Hz; and, Notch filter at 50 Hz. A galvanic isolation rated to 1,500 Vrms separated the MyoAmpF2F4-VGI8 from the power grid.

**Figure 6:**
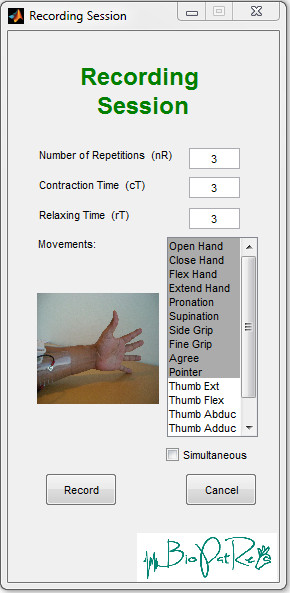
**Recording session.** Settings used for the recording session available in the repository: “10mov4chUntargetedForearm”.

Ten different hand and wrist movements were repeated 3 times during 3 seconds with equal relaxation periods between repetitions. The recording session settings are shown in Figure 6 as selected in the recording session GUI.

The selected movements were: open hand (OH), close hand (CH), flex hand (FH), extend hand (EH), pronation (PR), supination (SP), side grip (SG), fine grip (FG), agree or thumb up (AG), and pointer or index extension (PT). These movements were selected as they could be feasible in high-end commercial prostheses. Although recordings from amputee patients are not initially provided, it has been shown that algorithm comparisons hold between amputees and able-bodies, thus supporting the evaluation of such algorithms in the latter population [1]. It is worthy to keep in mind that a drop in classification accuracy between able bodies to amputees is expected [17], and that this difference should not be overlooked.

Most of the subjects used BioPatRec for the first time (82%) and only one subject had the electrodes placed in the dominant side. The average age was 31.1 (±11.1) years; 176 (±8) cm height; 68.3 (±11.8) kg weight; and 9 were females (53%). All subjects’ information is included in the recording sessions. None of the subjects had history of neuromuscular disorders. All subjects formally consent their participation in the experiment, as well as the publication of their recording session.

**Figure 7:**
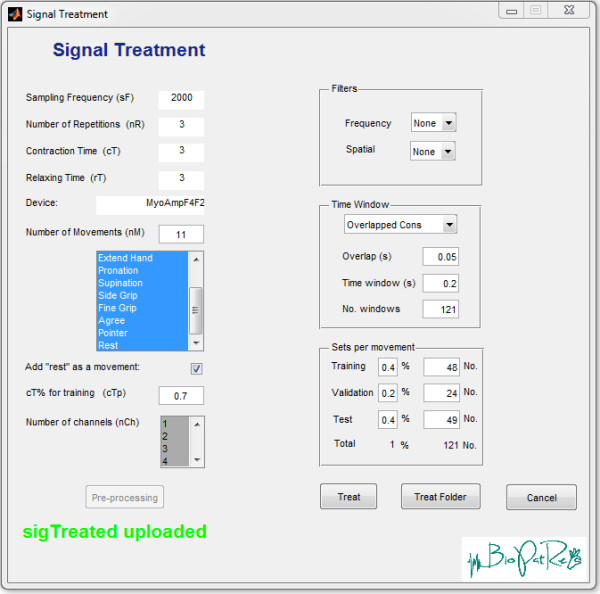
**Signal treatment.** Signal treatment settings used to compare the different classifiers.

This data set was used to compare the classification performance between RFN, ANN and LDA. All signal processing settings are shown in Figure 7. The recording sessions were treated with 0.7 cTp, that we have empirically found to be enough to partially conserve transient information (see Figure 2). The inclusion of the transient periods has been shown beneficial for real-time control, although it is known to decrease the offline accuracy of the classifier [40]. The “rest” position was added as an additional movement resulting in a classification task of 11 patterns. Overlapping windowing of 200 ms, with 50 ms time increment, was used as signal segmentation. It has been shown through information theory that EMG windows of 100 to 300 ms contain the highest information content [51]. Furthermore, optimal length for this specific task has been suggested to be between 150 and 250 ms [19,49].

In order to evaluate the classifiers offline performance, cross-validation of 100 trainings with randomized data sets were performed per subject and for each algorithm (1,700 per algorithm). The real-time performance was assessed using the motion test (3 trials, 3 repetitions, and 5 seconds timeout). Two subjects were excluded from the motion test due to constraints in their availability during the experiments. The order in which the classifiers were evaluated using the motion test was randomized between subjects. The most commonly used set of features (according to Table 1) was employed: mean absolute value, zero crossing, slope sign changes, and waveform length. The PC used was running 64-bits Windows 7 with processor at 3.1 GHz (Intel i3–2100), and 4 GB of RAM.

This study was approved by the Swedish Regional Ethics Committee in Gothenburg (626-10, T688-12).

### Statistical analysis

Since the origins of machine learning, different algorithms have been compared to each other over one or several data sets. A variety of tests for statistical significance have been applied, sometimes incorrectly, in order to justify the selection of the best performing algorithm [52]. Although few studies have compared several pattern recognition algorithms for prosthetic control, it is ANOVA [5,6,29], and Wilcoxon Signed-Rank [7] that have been used the most. In order to address the uncertainty of appropriate statistical tests, Demšar performed a thorough investigation on the topic concluding that the Wilcoxon Signed-Rank test is well suited for comparing pattern recognition algorithms on a single data set, and the Friedman test, with suitable post-hoc tests, when using data sets from different classification problems [52]. In this study, the statistical significance is evaluated using the Wilcoxon Signed Rank test at p<0.05, and values preceded by “ ±” represent the standard deviation.

## Results and discussion

### Regulatory feedback networks in prosthetic control

Table 2 summarizes the offline and real-time performance of each classifier. The time required for offline classification of all the testing sets was in average 1.03 (±0.018) ms, 0.58 (±0.003) ms, and 1.49 (±0.012) ms for LDA, MLP, and RFN respectively. These were all statistically significant differences. As expected, RFN had the slowest prediction speed since most of the algorithm itself is executed in the testing phase. Nevertheless, its corresponding prediction speed for a single input feature vector is still well suited for real-time control (2.76*μs*, considering the 49 sets per 11 movements). Furthermore, RFN has the lowest implementation complexity, thus making it suitable for stand-alone systems using microcontrollers.

**Table 2:** Offline and real-time results

	**Offline**				**Real-time**				
	**Classification of**	**Training**	**Accuracy**		**Selection**	**Completion**	**Completion**	**Accuracy**	
	**testing sets (ms)**	**time (s)**	**(%)**		**time (s)**	**time (s)**	**rate (%)**	**(%)**	
**LDA**	1.03 (±0.018)	0.125 (±0.002)	92.1 (±4)		0.622 (±0.24)	1.86 (±0.31)	87.3 (±11)	67.1 (±10)	
**MLP**	0.58 (±0.003)	164.1 (±52.06)	91.2 (±5)		0.807 (±0.27)	2.18 (±0.32)	75.8 (±13)	60.9 (±8.8)	
**RFN**	1.49 (±0.012)	0.552 (±0.007)	83.8 (±9)		0.627 (±0.22)	1.89 (±0.30)	78.0 (±12)	67.4 (±10)	

Performance summary of each classifier in predicting 11 movements in 17 subjects (offline) and 15 subjects (real-time).

The training and validation speed was 0.125 (±0.002) s, 164.1 (±52.06) s, and 0.552 (±0.007) s for LDA, MLP, and RFN respectively. All differences were statistically significant. It is worthy of notice that the validation time includes several testing loops which explains why RFN does not show the fastest training time although it requires no more than a simple average computation over all feature vectors of each class. As expected, the MLP required considerable longer training times in comparison with LDA and RFN.

**Figure 8:**
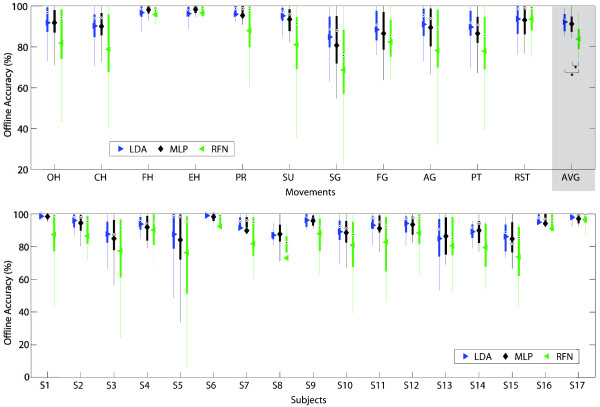
**Offline accuracy.** The offline accuracy between classifiers per movement (top) and subjects (bottom) are presented in box plots where the central mark represents the median value; the edges of the box are the 25th and 75th percentiles; the whiskers give the range of data values without considering outliers for clarity; and solid markers represent the mean. The average offline accuracy for LDA, MLP and RFN was 92.1(±0.04)%, 91.2(±0.05)%, and 83.5(±0.09)% respectively. Statistical significance (p<0.05) is shown only for the average values by “ * ”.

The overall offline accuracy for LDA, MLP and RFN was 92.1(±0.04)%, 91.2(±0.05)%, and 83.5(±0.09)% respectively. No statistical significance was found between LDA and MLP, but both were statistically significant against RFN. Figure 8 illustrates the comparison between movements and subjects.

Considerable variability was found between subjects, where the vast majority did not have any previous experience in this task. In contrast, the most experienced subject (S17) produced similar accuracies for all classifiers (>96%). Interestingly, the second best performing subject (S6), although unfamiliar with the task, is a professional musician presumably skilled in motor control, but more importantly, used to produce repetitive movements. It has been shown that practice helps to reduce the intra-class variability and therefore improvements can be achieved with subjects training [18]. This observation by Bunderson *et al.* is particularly relevant to RFN. The stability, or salience, of the RFN’s response is used to determine whether or not a given input is coherent with its representation in the connectivity matrix. Therefore, RFN is very dependent in a proper representation of each class by a single vector of features which would be obviously enhanced with lower intra-class variability.

**Figure 9:**
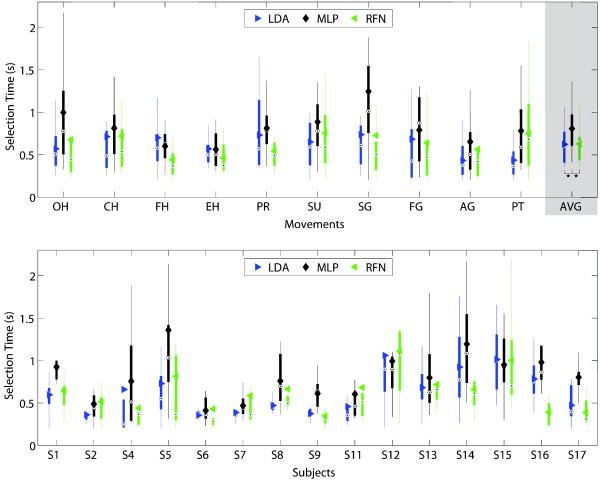
**Selection time The selection time between classifiers per movement (top) and subjects (bottom) are presented in box plots where the central mark represents the median value; the edges of the box are the 25th and 75th percentiles; the whiskers give the range of data values without considering outliers for clarity; and solid markers represent the mean.** The selection time reflects how fast the controller can produced the first correct prediction. It considers the time window (200 ms) and the time required for signal processing and classification. The average selection times for LDA, MLP and RFN were 0.62 (±0.24) s, 0.81 (±0.27) s, and 0.63 (±0.22) s, respectively. Statistical significance (p<0.05) is shown only for the average values by “ * ”.

**Figure 10:**
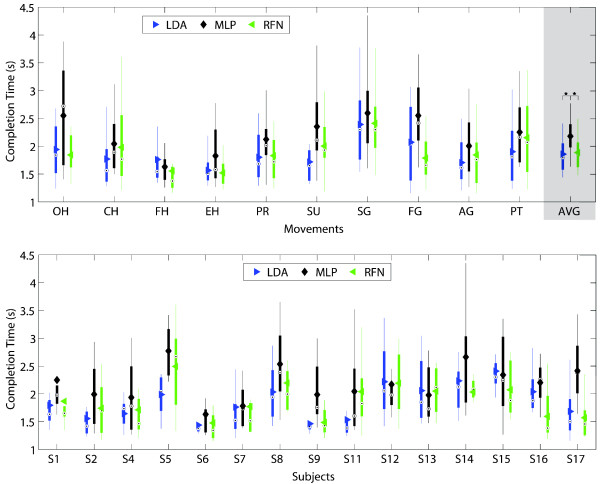
**Completion time.** The completion time between classifiers per movement (top) and subjects (bottom) are presented in box plots where the central mark represents the median value; the edges of the box are the 25th and 75th percentiles; the whiskers give the range of data values without considering outliers for clarity; and solid markers represent the mean. The completion times reflects the stability of the classifier by computing the time required for 20 correct predictions to occur. It considers a time window (200 ms), and the time required for signal processing, and classifier computation. The average completion times for LDA, MLP and RFN were 1.86 (±0.31) s, 2.18 (±0.32) s, and 1.89 (±0.30)s, respectively. Statistical significance (p<0.05) is shown only for the average values by “ * ”.

**Figure 11:**
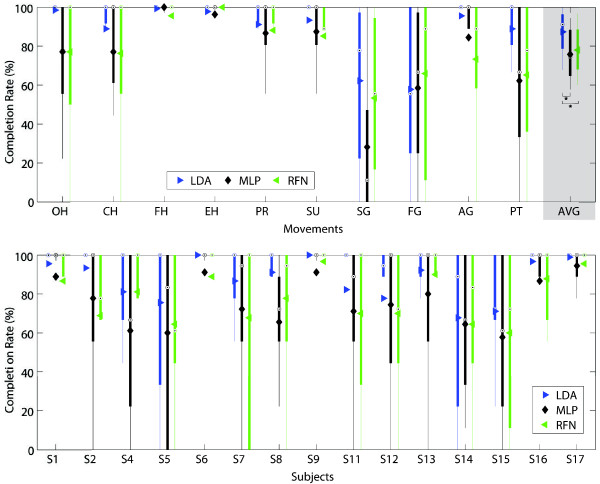
**Completion rate.** The completion rate between classifiers per movement (top) and subjects (bottom) are presented in box plots where the central mark represents the median value; the edges of the box are the 25th and 75th percentiles; the whiskers give the range of data values without considering outliers for clarity; and solid markers represent the mean. The completion rate is equal to the number of movements that achieved completion time over all the attempted movements. The average completion rates for LDA, MLP and RFN were 87.3(±11)%, 75.8(±13)%, and 78.0(±12)%, respectively. Statistical significance (p<0.05) is shown only for the average values by “ * ”.

**Figure 12:**
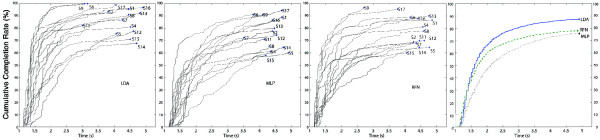
**Cumulative completion rate.** The cumulative completion rate illustrates the percentage of completed motions within a time span. E.g. The rightmost insert shows that over 80% of motions were completed within 3 seconds using LDA. Inserts from left to right show the cumulative completion rate of each trial per subject for LDA, MLP and RFN. The rightmost insert considers all trails of all subjects for each algorithm.

**Figure 13:**
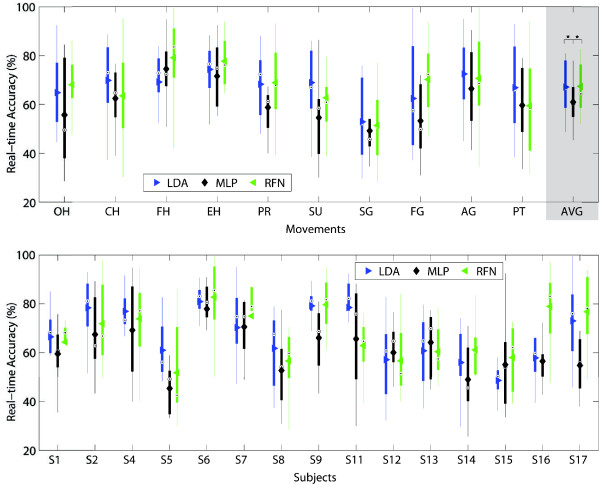
**Real-time accuracy.** The real-time accuracy between classifiers per movement (top) and subjects (bottom) are presented in box plots where the central mark represents the median value; the edges of the box are the 25th and 75th percentiles; the whiskers give the range of data values without considering outliers for clarity; and solid markers represent the mean. The real-time accuracy is computed by dividing the number of correct predictions during completion time over all predictions. If no motion completion was achieved, the accuracy was not considered. The real-time accuracies for LDA, MLP and RFN were 67.1(±10)%, 60.9(±8.8)%, and 67.4(±10)%, respectively. Statistical significance (p<0.05) is shown only for the average values by “ * ”.

**Figure 14:**
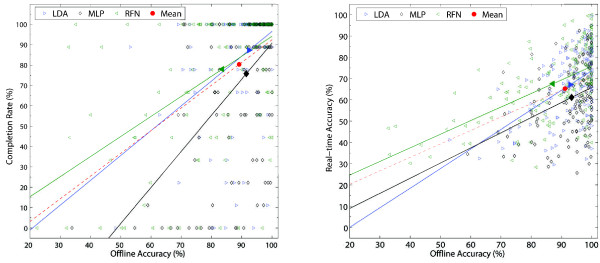
**Offline accuracy vs real-time indicators The offline accuracy per movement and subject is compared against their corresponding real-time accuracy and completion rate.** The mean of each classifier, and the mean of all three, are shown with solid markers. A linear fitting of the data is shown in continues lines per classifier, and for all data using a discontinuous line. The overall offline accuracy of 89.1% produced 80.4% completion rate (8.7% difference). An average offline accuracy of 91.2% was reduced to 65.3% real-time accuracy (25.9% reduction). The offline accuracy in the latter case only considered cases where the motion was completed in order to be paired with its corresponding real-time counterpart.

Figures 9, 10, 11, 12, 13 and 14 show the key performance indicators resulting from the motion tests. Although MLP has the fastest testing time (offline), its selection and completion times were slower than LDA and RFN. This can be explained by MLP’s low real-time accuracy (see Figure 13). In average, MLP made ∼40*%* misclassifications before reaching 20 correct predictions versus ∼30*%* from LDA and RFN.

The completion rate and its cumulative graphs (Figures 11 and 12) show a more consistent performance per movement and subjects for LDA, thus making it the best performing algorithm in this experiment. A weak relationship between offline accuracy and prosthetic controllability has been identified previously [8,17]. Figure 14 illustrates offline accuracy versus real-time indicators such as the completion rate and real-time accuracy. Contrasting results can be observed such as the high offline accuracies of LDA and MLP but considerably different real-time results. Conversely, RFN had around 10% lower offline accuracy than MLP but achieved similar completion rates, and notably, the best real-time accuracy. The latter suggests that RFN performs more consistently than LDA, and especially MLP, when considering their offline evaluation. It can be argued that when a proper representation of the class is given in the connectivity matrix, RFN produced the best results. This can be seen by examining the hand extentension and flexion movements (EH and FH), which had high offline accuracies and the fastest selection and completion times; the highest real-time accuracies; and, top completion rates. This would also explain RFN’s steeper slope at initial times of the overall cumulative completion rate (Figure 12). The introduction of a learning algorithm for RFN is thus advised, and it will be considered in a future study.

We have empirically experienced that high offline accuracy provides a false sense of high reliability, which translates into user frustration when the system does not behave as expected. RFN showed more consistency between offline and real-time performance, see Figure 14. In average, one to two movements had low offline accuracy which translated into an overall lower completion rate. However, the movements with higher accuracies normally performed as expected.

It has been suggested that classification accuracy over 90% normally yield a controllable system [53], while lower than 85% would not be acceptable for prosthetic control [1]. Our results show that estimating real-time performance from offline accuracy alone depends considerably on the algorithm in question, however, it can also be observed in subjects, and movements, that offline accuracies over 95% normally yielded over 90% completion rates.

A more practical implication of these results can be taken from the average reduction of ∼25*%* from offline to real-time accuracy, which motivates the use of post-processing techniques or control algorithms to compensate for this decay.

**Figure 15:**
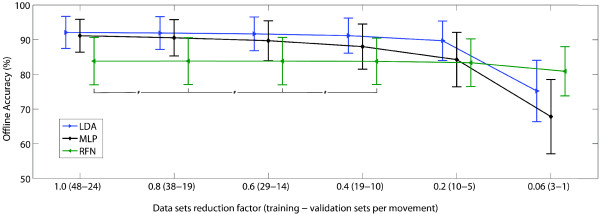
**The effect of decreasing the number of training and validations sets on offline accuracy.** The average accuracy and standard deviation of 100 trainings per each of the 17 subjects is shown for each classifier. The amount of available data sets was reduced from 100% to 6%, and the data sets were randomized before each training. The 100% represents 48 training and 24 validation sets, each a feature vector extracted from a 200 ms time window with 50 ms time increment. The testing sets were kept constant (49 per movement). A statistical significant reduction of accuracy was found between each step for LDA and MLP, but only for the last two steps for RFN. This suggests that RFN allows considerable reductions of training data while conserving similar classification accuracy. For clarity in the graph, only the non-statistical significant differences are shown by “ # ”.

RFN is a relatively simple but powerful algorithm that showed comparable results to those of more sophisticated classifiers such as MLP or LDA. The connectivity matrix was simply constructed using the average of the available feature vectors (“learning”), which in turns requires less information. Therefore, the training data can be decreased with little impact on the classification accuracy as shown in Figure 15. Conversely, a statistical significant reduction of accuracy was found while decreasing the information available for training the LDA and MLP classifiers. A shorter training requires less memory, which together with low computationally requirements, facilitates the implementation of RFN in stand-alone prosthetic systems based on microcontrollers.

### BioPatRec

**Figure 16:**
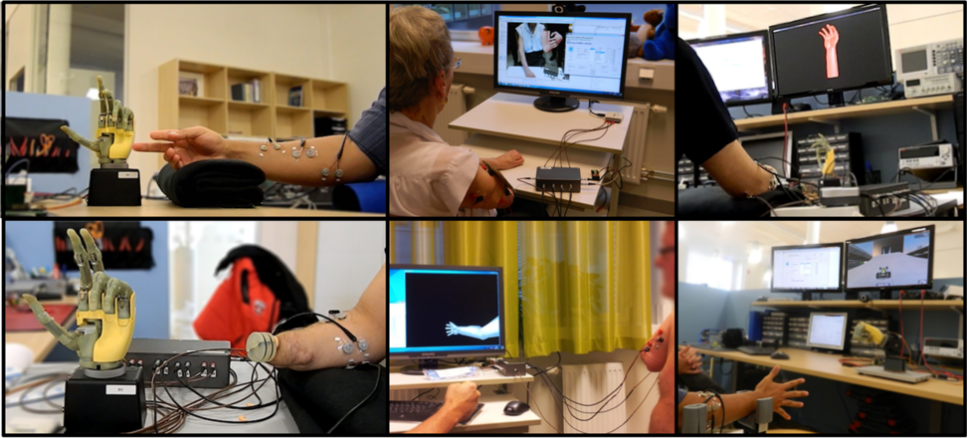
**Non-amputee and amputee subjects demonstrating BioPatRec applications.** The different insets in this figure show amputees and non-amputees using BioPatRec for the control of a multifunctional prosthesis; virtual limbs in augmented and virtual reality; and computer games. All these potential output possibilities from BioPatRec as a motion predicting software.

BioPatRec is demonstrated in this study by the implementation of a relatively new pattern recognition algorithm, namely Regulatory Feedback Networks (RFN). RFN was compared with two of the most popular classifiers in prosthetic control: LDA and MLP. The offline performance of LDA and MLP was found similar to previous comparisons [4-6], however, their real-time performance was unexpectedly different, thus supporting the need of real-time evaluations as those provided in BioPatRec. Additionally, videos demonstrating BioPatRec for the real-time control of a virtual limb and multifunctional prosthetic devices are available in the online project site [15]. Figure 16 shows ongoing applications of BioPatRec as an illustration of the possible outputs for the software.

BioPatRec has proven to be a research tool that facilitates international collaboration as it has been currently shared in three different continents (America, Europe and Australia). It has also promoted interest in prosthetic control among researchers and students from other disciplines (e.g. Artificial Intelligence, Medialogy, Augmented Reality, etc …). Furthermore, BioPatRec is used as a teaching tool for bio-electric signal processing and pattern recognition, as it provides real and practical examples suitable for problem-based learning. An updated list of the projects and collaborations around BioPatRec can be found online at [15].

### Future work

Although different sets of signal features can provide satisfactory results [49], an optimal selection has not yet been achieved. It has been suggested that the selection of features over classifiers has a higher impact on the classification performance [4,54]. Therefore algorithms for optimal feature selection are currently under implementation. A natural control of artificial limbs requires that different degrees of freedom can be controlled simultaneously [55]. Simultaneous control as well as different classifier topologies are currently explored and will be released in future versions of BioPatRec. A demonstration of simultaneous control is given in the project site [15].

The recording sessions are currently performed using the screen-guided training paradigm, which employs visual cues to indicate the patient when to execute which movement. This could be further improved by utilizing the VRE in a similar way as the prosthesis-guide training [56], where the user follows the artificial device while performing different movements.

## Conclusions

Signal processing and pattern recognition are important parts of the efforts devoted to improving the control of artificial limbs. In order to address specific research questions, research groups must develop their own dedicated software with considerably overlapping features. This results in a variety of algorithms and control strategies implemented in different platforms, which prevent direct comparison and the benefit of utilizing available knowledge as a starting point for further developments. BioPatRec provides a common research platform for prosthetic control strategies based in pattern recognition algorithms. It is released with all the necessary routines for the myoelectric control of a virtual hand and multifunctional prosthetic devices; from data acquisition to real-time evaluations. Moreover, it provides a shared repository of myoelectric signals useful for development, as well as for benchmarking on common data sets. Extensive documentation on its implementation is provided in the online hosting platform in order to ease utilization, speed up startups, and more importantly, promote collaboration from the different fields required in the multidisciplinary task of improving artificial limbs.

BioPatRec has been made open source with the hope to accelerate, through the contributions of the community, the development of better algorithms that can eventually improve the patient’s quality of life.

## Authors’ contributions

MOC programed BioPatRec, performed the algorithms comparison, and drafted the manuscript. RB and BH supervised this research and revised the manuscript. All the authors have read and approved the final manuscript.

## Competing interests

MOC was partially funded by and RB is a stockholder of Integrum AB, a medical device company developing bone-anchored prostheses. Originally intellectual property of Integrum AB, BioPatRec is released as open software to promote collaboration, and boost the development of advanced prosthetic control strategies. As dictated by the open source license, Integrum AB would benefit as much as any other individual, or commercial entity, from the developments made through BioPatRec.

## Supplementary Material

Additional file 1 **BioPatRec ETT: Summary of features.** Features of the first open source release version of BioPatRec: BioPatRec ETT. Click here for file
