# Supplementary material for: BioPatRec: A modular research platform for the control of artificial limbs based on pattern recognition algorithms
Source: Source Code Biol Med. 2013 Apr 18;8:11. doi: 10.1186/1751-0473-8-11 (PMC3669028; doi:10.1186/1751-0473-8-11)
Supplement: Additional file 1 — BioPatRec ETT: Summary of features. Features of the first open source release version of BioPatRec: BioPatRec ETT. [file 1751-0473-8-11-S1.PDF]

BioPatRec ETT: List of Features

Online at: [http://code.google.com/p/biopatrec]

August 20, 2012

1 General

- **Modular.** BioPatRec is organized in different modules that are linked through the use of structure arrays. These structure arrays can be saved and loaded between the different modules. This also allows replacing or modifying any module without affecting the others, given that the structure arrays are preserved.
- **Customizable.** As a result of the modular design, BioPatRec can be adapted to different experimental settings.
- **User friendly.** The use of graphical user interfaces (GUIs) facilitates BioPatRec utilization. Moreover, a considerable amount of documentation is available in the online hosting platform.

2 Recordings

- Dedicated GUI for customization of the recording sessions
- Dedicated GUI to load recording sessions, and display related information.
- Signals displayed in time and frequency domains.
- Graphical navigation tools available (time and frequency range selection, zoom, pan, etc. . .)
- Frequency and spatial filters available

3 Signal processing

- Dedicated GUI for signal processing
- Movements selection/filtering
- Channels selection/filtering
- Addition of “rest” as the state of “no movement”
- Filters
  - Frequency
  - Spatial
- Signal Segmentation
  - Non-overlapped with selectable *Time Window*
  - Constant overlap with selectable *Time Window* and *Overlap*
- Customizable construction of the training, validation and testing sets
- 27 time and frequency signal features available

4 Pattern Recognition

- Easy selection of signal features
- Customization of the training, validation and testing sets
- Pattern recognition and training algorithms:
  - Discriminant Analysis
    - Linear and diagonal linear
    - Quadratic and diagonal quadratic
    - Mahalanobis
  - Artificial Neural Networks - Multilayer Perceptron (MLP) trained by:
    - Back-propagation (BP)
    - Particle Swarm Optimization (PSO)
  - Regulation Feedback Networks (RFN) trained by:
    - Mean
    - Mean + PSO
    - Exclusive mean
- Different normalization methods:

  - Mean 0, Variance 1
  - 0 to 1
  - -1 to 1

- Optional randomization of data sets
- Display of accuracy per movement and confusion matrix
- Automatic computation of pattern recognition statistics
- Real-time evaluations:
  - Motion Test
  - Target Control Achievement

5 Control

- Control algorithms:
  - Major voting
  - Buffer output

6 Virtual Reality Environment

- Virtual lower arm with 7 degrees of freedom
